# Open versus Closed Kinetic Chain Exercises following an Anterior Cruciate Ligament Reconstruction: A Systematic Review and Meta-Analysis

**DOI:** 10.1155/2017/4721548

**Published:** 2017-08-17

**Authors:** Daniel Jewiss, Cecilia Ostman, Neil Smart

**Affiliations:** ^1^School of Rural Medicine, University of New England, Armidale, NSW 2351, Australia; ^2^School of Science and Technology, University of New England, Armidale, NSW 2351, Australia

## Abstract

**Background:**

There is no consensus on whether closed kinetic chain (CKC) or open kinetic chain (OKC) exercises should be the intervention of choice following an anterior cruciate ligament (ACL) injury or reconstruction.

**Methods:**

A systematic search identified randomized controlled trials of OKC versus CKC exercise training in people who had undergone ACL reconstructive surgery. All published studies in this systematic review were comparisons between OKC and CKC groups.

**Results:**

Seven studies were included. Lysholm knee scoring scale was not significantly different between OKC and CKC exercise patients: MD: −1.03%; CI: −13.02, 10.95; *p* value = 0.87 (Chi^2^  =  0.18, df = 1, and *p* value  =  0.67). Hughston clinic questionnaire scores were not significantly different between OKC and CKC exercise patients: MD: −1.29% (−12.02, 9.43); *p* value = 0.81 (Chi^2^  =  0.01, df = 1, and *p* value = 0.93).

**Conclusions:**

While OKC and CKC may be beneficial during ACL surgical rehabilitation, there is insufficient evidence to suggest that either one is superior to the other.

## 1. Introduction

Open kinetic chain (OKC) exercises are lower limb activities performed where the distal segment of the limb is free to move. The opposite of OKC is closed kinetic chain exercises (CKC). There is no consensus among the existing published evidence as to whether closed kinetic chain (CKC) or open kinetic chain (OKC) exercises should be the intervention of choice following an anterior cruciate ligament (ACL) injury or reconstruction. Several outcome measures are recommended to assess ACL injury and rehabilitation outcomes [1]. The commonly held belief has been that OKC exercises cause increased strain on the ACL as well as increased joint laxity and anterior tibial translation [2].

The doubts about the safety of OKC exercises are arguably unsupported by substantial published evidence and are possibly an intuitive opinion. Intuitively, a distally fixed foot in the case of CKC is safer than a nondistally fixed foot in OKC. This stance follows the work of Yack et al., 1993, who showed that there was greater joint laxity using the Anterior Tibial Displacement test during OKC exercises [3]. The assumption was thus made that OKC was more dangerous than CKC given that increased laxity is associated with graft failure and loosening. Beynnon and Fleming (1998) cast doubt as to whether measurable differences in strain between the two forms of exercise exist [4]. Many physiotherapists strongly believe that OKC exercises exert greater strain on the ACL and the patellofemoral joint than CKC exercises [4]. Moreover, the opinion of many physiotherapists is that adverse symptoms such as pain and joint laxity are more likely with OKC exercises than with CKC exercises.

We conducted a systematic analysis of all clinical randomized controlled trials comparing OKC exercises and CKC exercises in patients following ACL reconstruction. We aimed to determine whether there are any differences in clinical outcomes between OKC and CKC exercise protocols.

## 2. Methods

### 2.1. Search Strategy

Studies were identified through a MEDLINE search strategy (1966 to October 4, 2016), Cochrane Controlled Trials Registry (1966 to October 4, 2016), CINAHL, SPORTdiscus, and Science Citation Index. The search strategy included a mix of MeSH and free text terms for the key concepts related to anterior cruciate ligament reconstruction, exercise training, open chain exercises, and closed chain exercises (see PubMed search strategy in Supplementary Files available online at https://doi.org/10.1155/2017/4721548). Studies were included if patients had undergone ACL reconstruction. Searches were limited to prospective randomized or controlled trials in humans. No restrictions were placed on the language of publication. Reference list of papers and latest editions of relevant journals which were not available online were scrutinized for new references. Full articles were read and assessed by two reviewers (C. O. and D. J.) for relevance and study eligibility. Disagreements on methodology were resolved by discussion, and a third reviewer (N. S.) adjudicated over any disputes. Study authors were contacted and requested to provide further data if required.

### 2.2. Study Selection

Included studies were randomized controlled trials of OKC versus CKC exercise training in people who had undergone ACL reconstructive surgery. All published studies in this systematic review were comparisons between OKC and CKC groups.

In addition to the studies identified through database searching, reference lists of identified studies were scrutinized. Only the principal study with the greatest number of subjects was included where multiple publications existed from the same dataset. After initial screening we removed overlapping, duplicates, duplicate data, and irrelevant articles such as editorials and discussion papers that did not match the inclusion criteria. We excluded studies where the subjects had not yet undergone ACL reconstructive surgery, nonrelevant studies, and those reporting only acute exercise testing responses. We excluded studies from specific analyses if incomplete data was reported and the authors did not respond to our requests to provide missing data.

### 2.3. Outcome Measures

We extracted all possible data; however there were only two outcomes which were reported by more than one paper, the Lysholm knee scoring scale [1] and the Hughston clinic questionnaire [5]. We also recorded exercise training frequency, intensity, duration per session, length of exercise program, participant exercise adherence, and completion rates.

### 2.4. Data Synthesis

From extracted data we calculated the mean difference for pre/postintervention change in outcome measures and medical events.

### 2.5. Assessment of Study Quality

We assessed study quality with regard to eligibility criteria specific, random allocation of participants, concealed allocation, similarity of groups at baseline, assessors blinded, outcome measures assessed in 85% of participants, and intention of treatment analysis. The study quality was assessed according to the validated TESTEX scale which has a maximum score of 15 [6].

### 2.6. Data Synthesis

Meta-analyses were completed for continuous data by using the change in the mean and standard deviation of outcome measures. It is an accepted practice to only use postintervention data for meta-analysis but this method assumes that random allocation of participants always creates intervention groups matched at baseline for age, disease, severity, and so on. Change in postintervention mean was calculated by subtracting baseline from postintervention values. Data required was either (i) 95% confidence interval data for pre/postintervention change for each group or, when this was unavailable, (ii) actual *p* values for pre/postintervention change for each group or, if only the level of statistical significance was available, (iii) default *p* values; for example, *p* < 0.05 becomes *p* = 0.049, *p* < 0.01 becomes *p* = 0.0099, and *p*  =  not significant becomes *p* = 0.05.

### 2.7. Heterogeneity

Clinical heterogeneity was expected given that the data were obtained from patients represented in the included trials. As such, a random effects model was employed. Heterogeneity was quantified using the *I*^2^ test [7], as it does not inherently depend upon the number of studies considered. *I*^2^ values range from 0% (homogeneity) to 100% (greatest heterogeneity); a CI that does not include 0% indicates that the hypothesis of homogeneity is rejected, and an inference of heterogeneity is merited [7].

## 3. Results

**Figure 1:**
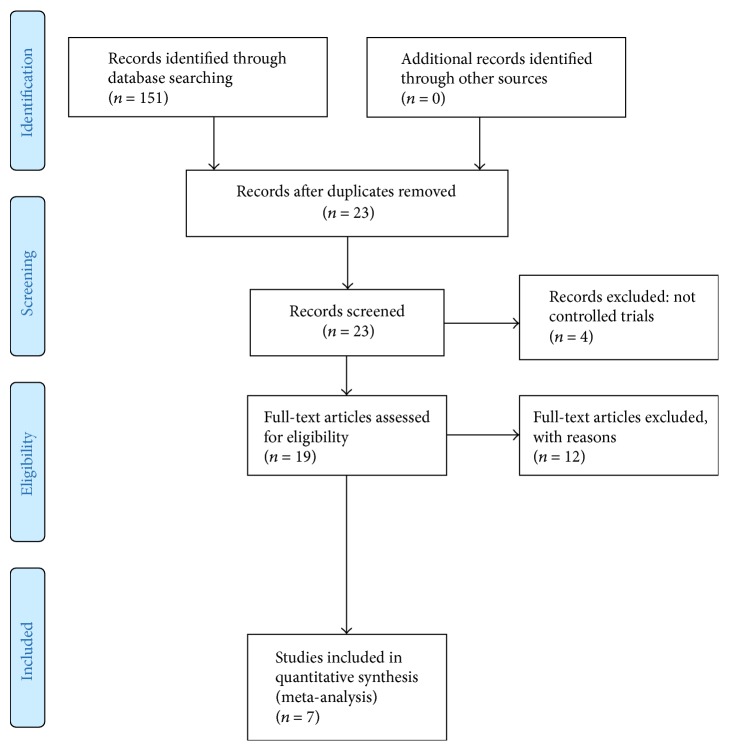
CONSORT statement.

Our initial search identified 151 studies. After excluding studies based on title and abstract, as well as removing duplicates, 23 studies remained. The full-text articles were assessed for study inclusion, and 16 were excluded (see Table 2); 4 studies were not randomized controlled trials, 6 studies were not postsurgical, and 6 studies did not compare OKC to CKC exercise rehabilitation. Seven studies remained, four were included for meta-analysis and three for systematic review only. The search details are provided in the CONSORT statement (Figure 1).

Our analysis of the 4 studies totalled 229 participants: 112 from OKC exercise groups and 117 from CKC exercise groups.  Table 1 summarizes the details of the included studies.

### 3.1. Meta-Analyses

#### 3.1.1. Lysholm Knee Scoring Scale

**Figure 2:**
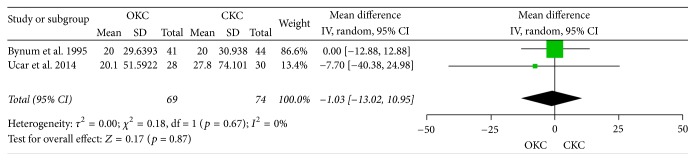
Lysholm score.

Two studies provided data on the Lysholm knee scoring scale. Results show that there was no significant change in OKC exercise patients versus CKC exercise patients: MD: −1.03% and CI: −13.02, 10.95; *p* value = 0.87 (Chi^2^  =  0.18, df  =  1, and *p* value = 0.67; between studies variability: *I*^2^ = 0%); see Figure 2.

#### 3.1.2. Hughston Clinic Questionnaire

**Figure 3:**
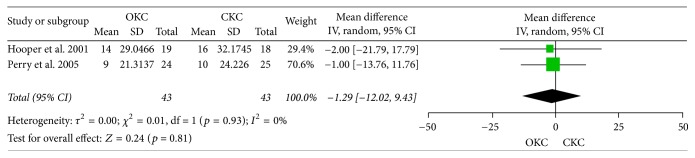
Hughston score.

Two studies provided data on the Hughston clinic questionnaire. Results show that there was no significant change in OKC exercise patients versus CKC exercise patients: MD: −1.29% (−12.02, 9.43); *p* value = 0.81 (Chi^2^  =  0.01, df = 1, and *p* value = 0.93; between studies variability: *I*^2^ = 0%); see Figure 3.

### 3.2. Systematic Review

Insufficient data existed to conduct meta-analyses for other outcome measures, so these are systematically described here.

#### 3.2.1. Patellofemoral Joint Pain

Although Bynum et al., 1995 [8], reported significantly less pain in CKC compared to OKC, they also reported no significant difference of Tegner activity scale score [9] in OKC versus CKC groups. Aside from varying levels of activity between groups, the authors also stated that these findings are not likely to be clinically significant. Ucar et al., 2014, reported more pain with OKC, but level of statistical significance was unclear [10]. Morrissey et al., 2000, found no significant difference between the groups in regard to knee extensor knee pain [11].

#### 3.2.2. Knee Extensor Strength

Kang et al., 2012, reported that isokinetic strength, isokinetic endurance, and squat strength improved significantly after both interventions (*p* < 0.05), but changes in isokinetic strength and isokinetic endurance of the extensor muscles were significantly greater in the OKC group than the CKC group (*p* < 0.05) [12]. In contrast Morrisey et al., 2000, found no difference between groups [11].

#### 3.2.3. Knee Anterior Laxity

Morrissey et al., 2000 [11], and Perry et al., 2005 [13], both found no statistically significant difference between OKC and CKC groups on Anterior Tibial Displacement test.

#### 3.2.4. Active Knee Flexion

Ucar et al., 2014, reported knee flexion was greater in the CKC group than the OKC group; however they do not report whether these differences were statistically significant [10].

### 3.3. Study Quality

The TESTEX scale [6] of study quality revealed a median score of 7 (out of a possible 14). As there was no sedentary control group in included studies TESTEX was scored out of 14 not usual 15. Study quality items that were not exhibited by more than 50% of studies were allocation concealment (1 study), assessor blinding (0 studies), intention of treatment analysis (0 studies), point measures and measures of variability reporting (3 studies), relative exercise intensity review (1 study), and exercise volume and expenditure (1 study).

## 4. Discussion

Our systematic review and meta-analysis suggest that a few direct, randomized, controlled comparisons of OKC and CKC knee extensor resistance training after anterior cruciate ligament reconstruction exist in the published literature. Both the statistical and descriptive analyses suggest both open and closed kinetic chain exercises are beneficial interventions for people with reconstructed ACLs. Moreover, of the few studies that do exist, our meta-analyses showed no significant difference between OKC and CKC for pain scores, knee extensor strength, laxity, and knee flexion.

### 4.1. Pain

Some previous work has asserted that CKC rehabilitative exercises are safer in terms of pain [14]. We also found evidence to the contrary [15]. Overall we found that there is insufficient evidence within the literature pool to either support or dispel this opinion. The meta-analyses of pain are unable to discern between OKC and CKC exercise rehabilitation programming. There are several explanations why no significant difference was seen. First, it may be that the questionnaire is not sensitive enough to detect small changes in pain and function. Second, the statistical power of the analyses is limited by a small number of studies with small sample size and the addition of future studies will determine if there is truly a difference. The final explanation is that there is actually no difference in pain experienced by people undertaking OKC versus CKC.

### 4.2. Strength

We were unable to pool data for an analysis of knee extensor strength. Descriptive analysis of the included studies of our systematic review was also inconclusive. A study of normal anterior cruciate ligament subjects was also unable to show either OKC or CKS to be superior for improving knee strength [16].

### 4.3. Knee Anterior Laxity

We were unable to pool data for analysis of knee extensor strength and descriptive analysis of included studies did not provide further clarity on superiority of either an OKC or CKC approach.

### 4.4. Knee Flexion

Only one study reported on the outcome measure knee flexion and this work claimed OKC exercise to be superior for restoring knee flexion after ACL reconstructive surgery. This work did not however provide statistical analyses to support the superiority of OKC. Further work on restoration of knee flexion after ACL surgery is clearly required.

The systematic review part of our work does appear to provide a stronger case for improvements in knee extensor strength with open kinetic chain exercises versus closed chain exercise. In contrast active knee flexion appears to be superior with closed kinetic chain exercises in comparison to open kinetic chain exercises. From our work, we cannot discern if patellofemoral joint pain and knee laxity are unaffected by the type of exercise rehabilitation used, shedding doubt as to whether OKC exercises are more likely to cause graft failure.

It is quite clear that the number of studies reporting meaningful data is currently limited. After conducting our search and analyses it is also apparent that the sample size of all studies is limited, with the exception of Bynum et al.'s 1995 study [8]. It is therefore surprising that such a strong contention exists that CKC exercises are optimal. Based upon the evidence presented in our work, one can but surmise that any preference for either OKC or CKC would be subjective at best. A large, well-designed, randomized, controlled trial is required to clarify this matter. There are significant advances in the diagnostic power of tissue imaging, such as Magnetic Resonance Imaging, since Bynum's 1995 work. The main purpose of MRI in patients with an ACL injury lies in accurate diagnosis or exclusion of a tear in patients with equivocal physical examination findings. ACL injury management is critically dependent on accurate diagnosis of other coexisting knee internal lesions, in particular tears of the lateral collateral ligament (LCL), posterior cruciate ligament (PCL), and the menisci [17]. It would also be prudent that future trials incorporate outcome measurements that are quantitative assessments, based upon measurements conducted by imaging experts who are blinded to participant allocations.

*Limitations*. The small volume of data precluded meta-analyses in several outcome measures and in those that were conducted statistical power was limited. Many of the outcome measures are self-reported subjective measurements. The existing literature has yet to optimize use of advances in imaging techniques. Study quality assessment suggests that future study designs could be more robust and strictly implement assessor blinding. Both of these enhancements would be likely to produce data more meaningful than those currently existing.

## 5. Conclusions

Based upon existing published data it is difficult to make a case for superiority of either open or closed chain kinetic exercise rehabilitation after ACL reconstruction. Meta-analyses failed to show a benefit of either type of exercise in terms of joint laxity and patellofemoral joint pain scores. While there was weak evidence that open chain exercises are better for improving knee extensor strength, this is countered by weak evidence for better active knee flexion in closed chain activities.

## Supplementary Material

PubMed Search Strategy.

## Figures and Tables

**Table 1:** Characteristics of included studies.

Author(s)	Participants	Country	Intervention	Weeks	Frequency (session/week)	Intensity	Session time (min)	Surgical approach	Outcomes
Bynum et al. 1995 [8]	97 patients randomized to OKC (*n* = 47) and CKC (*n* = 50).	US	Patients in the CKC group performed a mixture of resistance exercises including knee bends, seated leg press, stationary biking, and running. The OKC group performed a variety of exercises including leg raises, isotonic quadriceps with low weights, and treadmill jogging forwards and backwards.	52	Not provided	Not provided	Not provided	Arthroscopically assisted patellar tendon autograft and fixed with 9-mm interference screws.	Adverse events, graft failure, Lysholm knee function scoring scale, modified Tegner activity rating scale, overall patient assessment rating, patellofemoral pain, extension deficit, and flexion deficit using a KT-1000 (20 lb and max)

Hooper et al. 2001 [18]	37 patients randomized to OKC (*n* = 19) and CKC (*n* = 18).	UK	Patients in the CKC group performed unilateral resistance training of the hip and knee extensors on a leg press machine; patients in the OKC group performed exercises using knee and hip extension machines or ankle weights.	4	3	3 sets of 20 RM were done in each session for each training group. The training ROM for both hip and knee extensors in both groups was 90 to 0 degrees. Target speed settings were 1.5 s for the concentric phase and 3.0 s for the eccentric phase of training repetition, with a 1.0 s interval between phases.		Either arthroscopically assisted patellar tendon autograft or the technique described by Kennedy et al. (1980) using a fixed patellar tendon allograph.	Graft failure, Hughston clinic visual analog scale, knee flexion at heel-strike, peak extensor moment, extensor impulse, peak concentric power, and concentric energy.

Kang et al. 2012 [12]	36 patients were randomized to OKC (*n* = 18) and CKC (*n* = 18).	Korea	Stationary cycling for 5 minutes for warm-up and cool-down. OKC exercises were composed of straight leg raise, leg extension, and leg curl; CKC exercised were composed of squat, leg press, and lunge.	12	3	70% intensity of 1 repetition maximum (RM).	30 minutes	Not provided.	Extensor isokinetic strength, flexor isokinetic strength, extensor isokinetic endurance, flexor isokinetic endurance, and squat.

Morrissey et al. 2000 [11]	36 patients were randomized to OKC (*n* = 18) and CKC (*n* = 18)	UK	Patients in the CKC group performed unilateral resistance training of the hip and knee extensors on a leg press machine; patients in the OKC group performed exercises using knee and hip extension machines or ankle weights.	4	3	3 sets of 20 RM were done in each session for each training group. The training ROM for both hip and knee extensors in both groups was 90 to 0 degrees. Target speed settings were 1.5 s for the concentric phase and 3.0 s for the eccentric phase of training repetition, with a 1.0 s interval between phases.	Not provided	Either arthroscopically assisted patellar tendon autograft or the technique described by Kennedy et al. (1980) using a fixed patellar tendon allograph.	ADT

Morrissey et al. 2002 [15]	43 patients were randomized to OKC (*n* = 22) and CKC (*n* = 21).	UK	Patients in the CKC group performed unilateral resistance training of the hip and knee extensors on a leg press machine; patients in the OKC group performed exercises using knee and hip extension machines or ankle weights.	4	3	3 sets of 20 RM (rep max) were done in each session for each training group. The training ROM for both hip and knee extensors in both groups was 90 to 0 degrees. Target speed settings were 1.5 s for the concentric phase and 3.0 s for the eccentric phase of training repetition, with a 1.0 s interval between phases.	Not provided	Either arthroscopically assisted patellar tendon autograft or the technique described by Kennedy et al. (1980) using a fixed patellar tendon allograph.	Total cycling time, number of treatment sessions where patient was treated for pain/swelling, hypomobility, or poor balance/position sense, frequency for pain site location, Hughston clinic questionnaire (items 1, 2, and 25), knee extensor isometric peak torque, and knee pain during knee extensor isometric maximum peak torque.

Perry et al. 2005 [13]	49 patients randomized to CKC (*n* = 25) and OKC (*n* = 24).	UK	Patients in the CKC group performed unilateral resistance training of the hip and knee extensors on a leg press machine; patients in the OKC group performed exercises using knee and hip extension machines or ankle weights.	6	3	20 RM	Not provided	Either arthroscopically assisted patellar tendon autograft or the technique described by Kennedy et al. (1980) using a fixed patellar tendon allograph.	Hughston clinic questionnaire, training parameters, ATD, horizontal jump, vertical jump, and crossover jump.

Ucar et al. 2014 [10]	66 patients were randomized to CKC (*n* = 33) and OKC (*n* = 33).	Turkey	Patients in the CKC group performed squatting lunges, standing weight shift, wall sits, one-legged quad dips, and lateral step-ups; patients in the OKC group performed isometric quadriceps, flexor-extensor bench, isotonic quadriceps, long leg press on-off, and knee flexion-extension stretching exercises.	Not provided	Not provided	Not provided	Not provided	Arthroscopically assisted hamstring autograft.	Subjective pain visual analog scale, thigh circumference, knee flexion, and Lysholm score.

**Table 2:** Excluded studies.

Study	Reason for exclusion
Bird and Bulkeley 2010 [19]	Review paper
Chrzan et al. 2013 [20]	Intervention was not OKC versus CKC
Davis 1996 [21]	Population was not postsurgical
Dolan 2010 [22]	Not an RCT
Fitzgerald 1997 [23]	Not an RCT
Hooper et al. 2002 [24]	Intervention was not OKC versus CKC
Jenkins et al. 1997 [25]	Population was not postsurgical
Keays et al. 2013 [26]	Population was not postsurgical
Laboute et al. 2008 [27]	Intervention was not OKC versus CKC
Lage et al. 1995 [28]	Population was not postsurgical
Mikkelsen et al. 2000 [29]	Intervention was not OKC versus CKC
Neeter et al. 2006 [30]	Intervention was not OKC versus CKC
Perry et al. 2005 [13]	Population was not postsurgical
Petschnig and Baron 1997 [31]	Intervention was not OKC versus CKC
Rennison 1996 [32]	Not an RCT
Ross et al. 2001 [33]	Not an RCT
Tagesson et al. 2008 [34]	Population was not postsurgical
