# Supplementary material for: Open versus Closed Kinetic Chain Exercises following an Anterior Cruciate Ligament Reconstruction: A Systematic Review and Meta-Analysis
Source: J Sports Med (Hindawi Publ Corp). 2017 Aug 17;2017:4721548. doi: 10.1155/2017/4721548 (PMC5585614; doi:10.1155/2017/4721548)
Supplement: Supplementary file 1 — PubMed Search Strategy. [file 4721548.f1.docx]

**Supplementary Files**

PubMed Search Strategy

| [#](http://www.ncbi.nlm.nih.gov/pubmed/?querykey=8&dbase=pubmed&querytype=eSearch&)11 | | Search (Exercise/Broad[filter]) AND (#10) |  |
| --- | --- | --- | --- |
| [#](http://www.ncbi.nlm.nih.gov/pubmed/?querykey=6&dbase=pubmed&querytype=eSearch&)10 | | Search #8 OR #9 |  |
| [#](http://www.ncbi.nlm.nih.gov/pubmed/?querykey=5&dbase=pubmed&querytype=eSearch&)9 | | Search #1 OR #2 OR #3 OR #4 OR #5 OR #6 OR #7 OR #8 |  |
| [#](http://www.ncbi.nlm.nih.gov/pubmed/?querykey=4&dbase=pubmed&querytype=eSearch&)8 | | Search (((exercise[MeSH Terms]) OR exercise therapy [MeSH Terms]) OR aerobic exercise[Text Word]) OR exercise training[Text Word] OR resistance training [Text Word] OR Weight lifting [Text word] OR Strength Endurance [Text Word] OR Fitness [Text Word] OR rehabilitation [Text Word] OR Muscular exercise [Text Word] |  |
| [#](http://www.ncbi.nlm.nih.gov/pubmed/?querykey=3&dbase=pubmed&querytype=eSearch&)7 | | Search ((anterior cruciate ligament [MeSH Terms]) OR surgey [Text Word]) |  |
| [#](http://www.ncbi.nlm.nih.gov/pubmed/?querykey=2&dbase=pubmed&querytype=eSearch&)6 | | Search ((((anterior cruciate ligament [MeSH Terms]) OR injury [Text Word]) |  |
| [#](http://www.ncbi.nlm.nih.gov/pubmed/?querykey=1&dbase=pubmed&querytype=eSearch&)5 | Search (((((anterior cruciate ligament [MeSH Terms]) AND reconstruction [Text Word]) | |  |
| [#](http://www.ncbi.nlm.nih.gov/pubmed/?querykey=1&dbase=pubmed&querytype=eSearch&)4 | Search (((((anterior cruciate ligament [MeSH Terms]) OR exercise training [Text Word]) | |  |
| #3 | Search (((((anterior cruciate ligament [MeSH Terms]) OR closed kinetic chain [Text Word]) | | |
| [#](http://www.ncbi.nlm.nih.gov/pubmed/?querykey=1&dbase=pubmed&querytype=eSearch&)2 | Search (((((anterior cruciate ligament [MeSH Terms]) OR open kinetic chain [Text Word]) | | |
| [#1](http://www.ncbi.nlm.nih.gov/pubmed/?querykey=1&dbase=pubmed&querytype=eSearch&) | Search (((((anterior cruciate ligament [MeSH Terms]) AND rehabilitation [Text Word]) | | |
